# Gamete-Type Dependent Crossover Interference Levels in a Defined Region of *Caenorhabditis elegans* Chromosome V

**DOI:** 10.1534/g3.113.008672

**Published:** 2013-11-15

**Authors:** Idan Gabdank, Andrew Z. Fire

**Affiliations:** * Department of Pathology, Stanford University School of Medicine, Stanford, California 94305; † Department of Genetics, Stanford University School of Medicine, Stanford, California 94305

**Keywords:** recombination, crossover interference, meiosis, *Caenorhabditis elegans*

## Abstract

In certain organisms, numbers of crossover events for any single chromosome are limited (“crossover interference”) so that double crossover events are obtained at much lower frequencies than would be expected from the simple product of independent single-crossover events. We present a number of observations during which we examined interference over a large region of *Caenorhabditis elegans* chromosome V. Examining this region for multiple crossover events in heteroallelic configurations with limited dimorphism, we observed high levels of crossover interference in oocytes with only partial interference in spermatocytes.

Despite their occurrence in several systems, interference phenomena in which one crossover event on a chromosome reduces the probability of a second crossover have remained something of a mystery (Berchowitz and Copenhaver 2010). *Caenorhabditis elegans* provides an outstanding model for meiotic regulation and a system of choice to study detailed genetic properties of meiosis (Zetka 2009). Although interference certainly occurs in *C. elegans* (Hodgkin *et al.* 1979), literature reports tend to differ regarding levels of interference depending on age, temperature, gender, linkage group, and experimental design. Several individual observations are described below:

- A complete or near-complete lack of double crossovers is seen on the X chromosome in oocytes (Hodgkin *et al.* 1979; Hayashi *et al.* 2010).
- Hodgkin *et al.* (1979) show double crossovers during spermatogenesis in a region of LGIV, albeit with ∼55% interference (Hodgkin *et al.* 1979).
- Zetka and Rose (1995) find double crossover in males in a region of LGI at a frequency that suggested little or no interference during spermatogenesis (Zetka and Rose 1995).
- Meneely *et al.* (2002) fail to detect double crossovers during spermatogenesis in a region of LGIII (Meneely *et al.* 2002).
- Analyses of double crossover frequencies detected in the case of fused chromosomes performed by Hillers and Villenueve (2003) suggest that the *C. elegans* crossover control mechanism recognizes a DNA unit defined by a region capable of continuous homologous synapsis (Hillers and Villeneuve 2003).
- Nabeshima *et al.* (2004) report almost complete crossover interference in oocytes on chromosomes I and X (Nabeshima *et al.* 2004).
- Henzel *et al.* (2011) found crossover distribution along chromosome IV portion of mnT12 (fusion of IV and X chromosomes) to be significantly different between males and hermaphrodites. Their findings in males indicate that crossovers in adjacent chromosome intervals are discouraged, with the strength of crossover interference diminished by distance (Henzel *et al.* 2011).
- Hammarlund *et al.* (2005) report essentially complete interference for both oogenesis and spermatogenesis in hermaphrodite animals, on chromosomes V and X in spite of a large homology gap created by a heterozygous large insertion. The homology gap apparently alters crossover distribution but not the interference (Hammarlund *et al.* 2005).
- A number of studies provide data consistent with differential regulation of crossover interference in the two gamete lines (oocytes and sperm) (Howell *et al.* 1987; K. S. McKim and M. C. Zetka unpublished results cited in Zetka and Rose 1995; Lim *et al.* 2008).

In addition to differences in the region assayed for interference, we noted that the diverse nature of experimental designs somewhat limits direct comparisons between studies. Some studies used strains in which extensive polymorphic differences between chromosomes could conceivably affect pairing dynamics, whereas others used strains with known differences limited to small numbers of experimentally induced mutant alleles. Other growth and assay conditions also differed between studies in this area (including growth temperature, which is known to affect recombination frequencies in *C. elegans*) (Lim *et al.* 2008). We describe crossover interference levels in oogenesis and spermatogenesis observed using three widely spaced markers on *C. elegans* LGV (Figure 1A).

**Figure 1:**
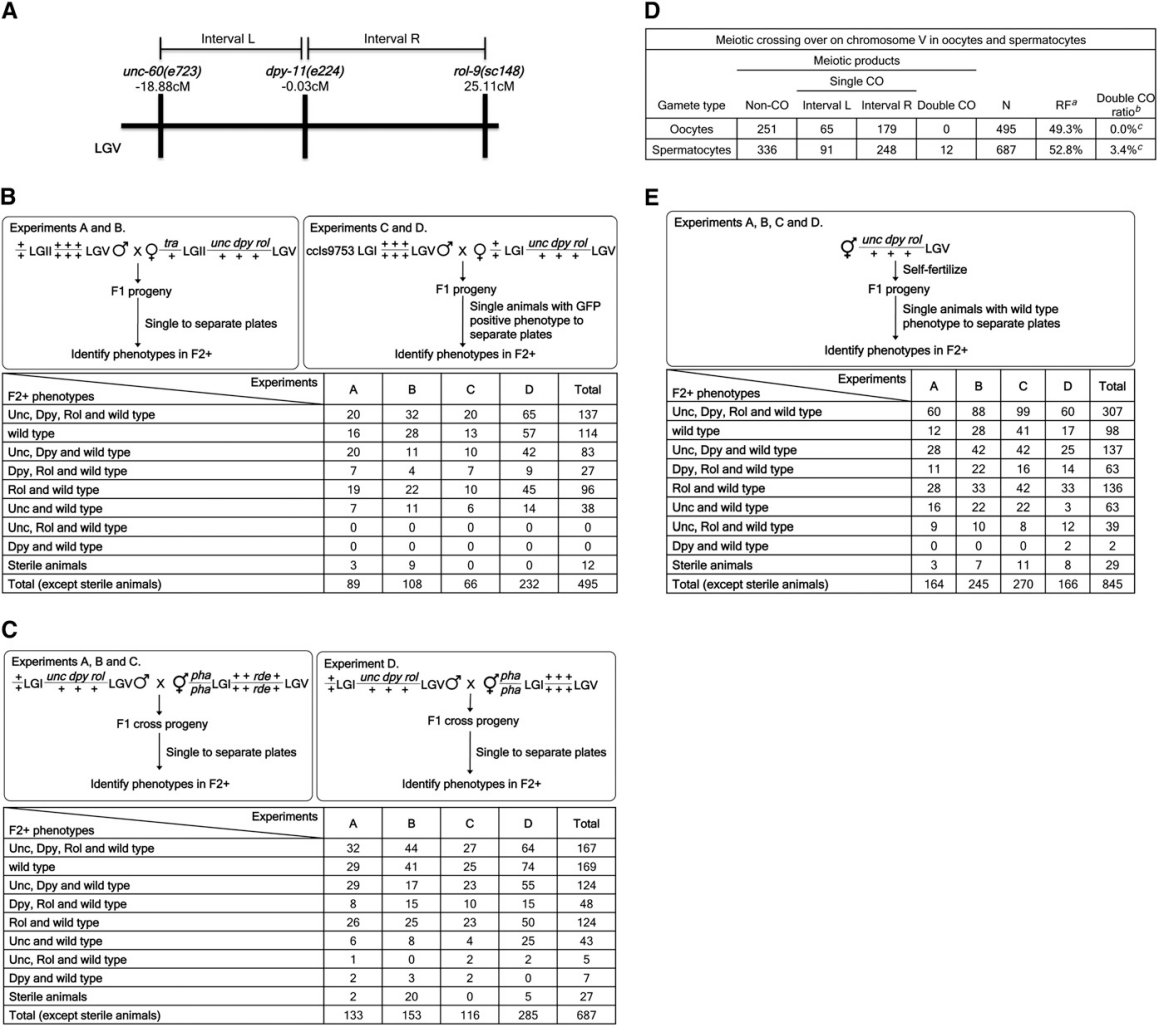
(A) Map positions of the genetic markers used in crossover assays. Schematic diagram indicates the markers used in recombination analysis and their corresponding genetic map positions. The markers used and the intervals assessed (L, R) for the three-marker analysis are indicated above the map. Map positions given in the figure are from Wormbase (Harris *et al.* 2010). (B) Crossover frequencies in oogenesis. Table entries are counts of F1 genotypes from each cross, as detected by examination of F1 progeny. Experiments A, B, and D were conducted at 23°, and experiment C was conducted at 16°. *tra* = *tra-2(q122gf)*; *unc* = *unc-60(e723)*; *dpy* = *dpy-11(e224)*; *rol* = *rol-9(sc148)*; ccIs9753, integration on chromosome I of *myo2*::*gfp*, *pes-10*::*gfp* and *gut*::*gfp*. (C) Crossover frequencies in spermatogenesis. Table entries are counts of F1 genotypes from each cross, as detected by examination of F1 progeny. Experiments A, B, and D were conducted at 23°, and experiment C was conducted at 25°. *rde* = *rde-1(ne300)*; *unc* = *unc-60(e723)*; *dpy* = *dpy-11(e224)*; *rol* = *rol-9(sc148)*; *pha* = *pha-1(e2123ts)*. (D) Meiotic crossing-over on chromosome V in oocytes and spermatocytes. *^a^*[(Total number of crossover events)/(number of meiotic products analyzed)] × 100. *^b^*[(Number of double crossovers)/(number of single crossovers + number of double crossovers)] × 100. *^c^*Oocytes and spermatocytes differed significantly with respect to the relative incidence of double crossovers *vs.* single crossovers among meiotic products with crossover. Fisher exact test for independence p-value = 0.002. (E) Crossover frequencies in self-fertilization. Table entries are counts of F1 genotypes resulting from the self-fertilization, as detected by examination of F1 progeny. All experiments were conducted at 23°. *unc* = *unc-60(e723)*; *dpy* = *dpy-11(e224)*; *rol* = *rol-9(sc148)*.

## Materials and Methods

Mapping markers LGV: *unc-60(e723)*, *dpy-11(e224)*, *rol-9(sc148)* Used for production of feminized animals LGII: *tra-2(q122gf)* Other mutations present (in post-recombination tester strains) LGI: ccIs9753(*myo2*::*gfp*, *pes-10*::*gfp*, *gut*::*gfp*) LGIII: *pha-1(e2123ts)* LGV: *rde-1(ne300)*.

Standard methods were used for growing and handling animals. Homozygous triple-mutants having *unc-60(e723)*, *dpy-11(e224)*, and *rol-9(sc148)* mutations on LGV were generated (Brenner 1974) and used to measure the crossover frequency in the experiments described. Crossover interference values are presented as [1−(observed double crossover events)/(expected double crossover events)]×100.

We note the formal possibility that a fraction of apparent double crossovers may actually reflect gene conversion events. An upper bound on gene conversion for *C. elegans* can be deduced from two-point mapping data (Moerman and Baillie 1979; McKim *et al.* 1988), with data relevant to the *dpy-11* lesion *e224* available from Rosenbluth *et al.* (1988). In all cases, gene conversions can be inferred to represent a sufficiently rare category of events to be negligible in our interference analysis.

### Oogenesis double-crossover frequency

To measure recombination frequency in oogenesis, we performed two parallel experiments with a similar experimental outline. First, a cross was performed between males with a wild-type chromosome V and *unc-60(e723) dpy-11(e224) rol-9(sc148)* V heterozygous triple-mutant hermaphrodites. Progeny were singled onto separate plates. Genotypes of the animals resulting from this cross were determined through subsequent self-fertilization and examination of subsequent generations for appearance of Unc, Dpy, and Rol phenotypes. Selection of cross progeny in the first generation cross is critical for interpretation of the gametic source of recombination.

In a first set of experiments, the heterozygous triple-mutant *unc-60(e723) dpy-11(e224) rol-9(sc148)/+++* were crossed with males having integrated GFP markers on chromosome I, allowing specific selection of the cross progeny. Cross-progeny animals were singled onto separate plates and their progeny were used to quantify different crossover events. Note in this case that introduction of the transgene postdates the inferred recombination events.

The second set of experiments initiated from a cross of homozygous triple-mutants to a *tra-2(q122gf)* males resulted in a population of heterozygous XX females carrying the feminizing mutation *tra-2(q122gf)*. Because these hermaphrodites are not able to self-fertilize because of the *tra-2(q122gf)* mutation, a subsequent cross with wild-type (N2) males resulted in only cross-progeny animals. Cross-progeny animals were singled onto separate plates and their progeny were used to identify parental genotypes and thus to quantify recombination frequencies. Singled animals carrying the *tra-2(q122gf)* mutation on chromosome II were discarded because of their inability to self-fertilize.

### Male spermatogenesis double-crossover frequency

To quantify double-crossover frequency in male spermatogenesis, we crossed *unc-60(e723) dpy-11(e224) rol-9(sc148)/+++* heterozygous triple-mutant males to *pha-1(e2123ts)* hermaphrodites at the nonpermissive temperature. Homozygous *pha-1(e2123ts)* embryos failed to survive growth at temperatures more than 23°, ensuring survival of only cross progeny. Cross-progeny animals were singled onto separate plates, with occurrence of phenotypes in subsequent generations used to determine genotypes and to detect crossover events.

### Self-fertilization double-crossover frequency

To quantify double crossovers in self-fertilization, we allowed *unc-60(e723) dpy-11(e224) rol-9(sc148)/+++* heterozygous triple-mutant animals to self-fertilize and then singled to separate plates their phenotypically wild-type progeny. Although not all double-crossover events can be detected (because of the potential for recombinant chromosomes originating from both gametes), one of the genotypes (*dpy-11(e224)/+*) can only be produced through a double-crossover–type event.

### Confidence Intervals

The confidence intervals (C.I.s) were calculated using the Clopper and Pearson exact method for binomial proportion (Clopper and Pearson 1934).

## Results

Measurements of interference entail a comparison of the product of individual two-point recombination frequencies between markers with the corresponding double-crossover frequency. We used standard genetic crosses to measure the frequency of single and multiple crossover events. Because *C. elegans* is a hermaphroditic species, we assayed frequencies in hermaphrodite oogenesis, male spermatogenesis, and in an aggregate measure that combines hermaphrodite spermatogenesis and oogenesis (Figure 1, B, C, and E, respectively).

Figure 1B presents the set of results observed in oogenesis. The observed oocyte two-factor recombination frequencies in our experiments were 13.1% [in agreement with results reported by Hammarlund *et al.* (2005)] and 36.2%, respectively, for the *unc-60(e723)*-to-*dpy-11(e224)* (interval L) and *dpy-11(e224)*-to-*rol-9(sc148)* (interval R) intervals (95% C.I.s, 10.3%–16.42% and 31.9%–40.6%, respectively) (Clopper and Pearson 1934). In the absence of crossover interference, one would expect 4.75% double recombinants in our experimental setup. Given 495 examined animals, 24 double recombinants would have been expected. We detected no double recombinants in oocyte meiosis, indicating high crossover interference (95% C.I., 87.5%–100% crossover interference).

Figure 1C presents results obtained in male spermatogenesis. The observed male spermatocyte two-factor recombination frequencies in our experiments were 15.0% and 37.8%, respectively, for the intervals L and R (95% C.I.s, 12.4%–17.9% and 34.2%–41.6%, respectively). The 12 cases of double crossovers in a population of 687 animals suggested partial, but not complete, crossover interference in the range of 69.2% (95% C.I., 48.7%–84.6% interference).

This analysis provides evidence for gender-specific interference effects. Despite comparable levels of total recombination (49.3% and 52.8%, respectively, for the assayed portion of chromosome V), we found an extreme difference in proportion of double-crossover events with double-crossover products not detected in oocyte meiosis while accounting for 3.4% of total crossover products from male spermatocyte meiosis. The difference between oocytes and male spermatocytes is statistically significant, with p-value of 0.002 (Figure 1D).

In these experiments, the interrogated oocytes and sperm differ in both cell biology and chromosome content (oocytes were from 2X:2A animals, sperm were from 1X:2A animals). An additional self-fertilization experiment was performed to assess double crossovers in XX sperm. As diagrammed in Figure 1E, we could unambiguously distinguish a class of double-crossover events from self-fertilization. Assuming complete crossover interference in oogenesis and 69.2% interference in spermatogenesis, the number of expected double recombinants that could be detected in our approach in the examined population of 845 animals would be three. In our self-fertilizing experiment, we have detected two double-recombinant animals. Because the previous outcross experiments had ruled out substantial double-crossover events during XX hermaphrodite oogenesis, we inferred that the self-fertilization–observed double crossovers were likely to have occurred during hermaphrodite (XX) spermatogenesis.

## Conclusion

The use of distant genetic markers on LGV allowed us to monitor double crossovers and interference intensity in oogenesis and spermatogenesis. We found interference for LGV during oogenesis to be very high (with the observation of zero detected double recombinants among 495 sampled meioses compared to an expectation of 24 for no interference), suggesting complete or at least near-complete interference. Crossover interference during male spermatogenesis was not complete, with 12 double-recombinant animals detected among 687 sampled (expected number for no interference, 39; interference, 69.2%). The cytological mechanism underlying the dichotomy of high interference in oogenesis and lower interference in spermatogenesis remains a question for future work.

## References

[bib1] Berchowitz L. E. Copenhaver G. P., 2010 Genetic interference: Don’t stand so close to me. Curr. Genomics 11: 91–102. 20885817 10.2174/138920210790886835 PMC2874225

[bib2] Brenner S., 1974 The genetics of *Caenorhabditis elegans*. Genetics 77: 71–94. 4366476 10.1093/genetics/77.1.71 PMC1213120

[bib3] Clopper C. J. Pearson E. S., 1934 The use of confidence or fiducial limits illustrated in the case of the binomial. Biometrika 26: 401–413.

[bib4] Hammarlund M. Davis M. W. Nguyen H. Dayton D. Jorgensen E. M., 2005 Heterozygous insertions alter crossover distribution but allow crossover interference in *Caenorhabditis elegans*. Genetics 171: 1047–1056. 16118192 10.1534/genetics.105.044834 PMC1456811

[bib5] Harris T. W. Antoshechkin I. Bieri T. Blasiar D. Chan J., 2010 WormBase: a comprehensive resource for nematode research. Nucleic Acids Res. 38(Database issue): D463–D467. 19910365 10.1093/nar/gkp952 PMC2808986

[bib6] Hayashi M. Mlynarczyk-Evans S. Villeneuve A. M., 2010 The synaptonemal complex shapes the crossover landscape through cooperative assembly, crossover promotion and crossover inhibition during *Caenorhabditis elegans* meiosis. Genetics 186: 45–58. 20592266 10.1534/genetics.110.115501 PMC2940310

[bib7] Henzel J. V. Nabeshima K. Schvarzstein M. Turner B. E. Villeneuve A. M., 2011 An asymmetric chromosome pair undergoes synaptic adjustment and crossover redistribution during *Caenorhabditis elegans* meiosis: Implications for sex chromosome evolution. Genetics 187: 685–699. 21212235 10.1534/genetics.110.124958 PMC3063665

[bib8] Hillers K. J. Villeneuve A. M., 2003 Chromosome-wide control of meiotic crossing over in *C. elegans*. Curr. Biol. 13: 1641–1647. 13678597 10.1016/j.cub.2003.08.026

[bib9] Hodgkin J. Horvitz H. R. Brenner S., 1979 Nondisjunction mutants of the nematode *Caenorhabditis elegans*. Genetics 91: 67–94. 17248881 10.1093/genetics/91.1.67 PMC1213932

[bib10] Howell A. M. Gilmour S. G. Mancebo R. A. Rose A. M., 1987 Genetic analysis of a large autosomal region in *Caenorhabditis elegans* by the use of a free duplication. Genet. Res. 49: 207–213.

[bib11] Lim J. G. Y. Stine R. R. W. Yanowitz J. L., 2008 Domain-specific regulation of recombination in *Caenorhabditis elegans* in response to temperature, age and sex. Genetics 180: 715–726. 18780748 10.1534/genetics.108.090142 PMC2567375

[bib12] Meneely P. M. Farago A. F. Kauffman T. M., 2002 Crossover distribution and high interference for both the X chromosome and an autosome during oogenesis and spermatogenesis in *Caenorhabditis elegans*. Genetics 162: 1169–1177. 12454064 10.1093/genetics/162.3.1169 PMC1462340

[bib13] McKim K. S. Heschl M. F. P. Rosenbluth R. E. Baillie D. L., 1988 Genetic organization of the *unc-60* region in *Caenorhabditis elegans*. Genetics 118: 49–59. 8608931 10.1093/genetics/118.1.49 PMC1203265

[bib14] Moerman D. G. Baillie D. L., 1979 Genetic organization in *Caenorhabditis elegans*: fine-structure analysis of the *unc-22* gene. Genetics 91: 95–103. 17248882 10.1093/genetics/91.1.95 PMC1213933

[bib15] Nabeshima K. Villeneuve A. M. Hillers K. J., 2004 Chromosome-wide regulation of meiotic crossover formation in *Caenorhabditis elegans* requires properly assembled chromosome axes. Genetics 168: 1275–1292. 15579685 10.1534/genetics.104.030700 PMC1448768

[bib16] Rosenbluth R. E. Rogalski T. M. Johnsen R. C. Addison L. M. Baillie D. L., 1988 Genomic organization in *Caenorhabditis elegans*: deficiency mapping on linkage group V(left). Genet. Res. 53: 105–118.

[bib17] Zetka M. C., 2009 Homologue pairing, recombination and segregation in *Caenorhabditis elegans*. Genome Dyn. 5: 43–55. 18948706 10.1159/000166618

[bib18] Zetka M. C. Rose A. M., 1995 Mutant *rec-1* eliminates the meiotic pattern of crossing over in *Caenorhabditis elegans*. Genetics 141: 1339–1349. 8601478 10.1093/genetics/141.4.1339 PMC1206871

